# Supplementary material for: Overcoming mcr-1 mediated colistin resistance with colistin in combination with other antibiotics
Source: Nat Commun. 2018 Jan 31;9:458. doi: 10.1038/s41467-018-02875-z (PMC5792607; doi:10.1038/s41467-018-02875-z)
Supplement: Supplementary file 2 — Supplementary Information [file 41467_2018_2875_MOESM2_ESM.pdf]

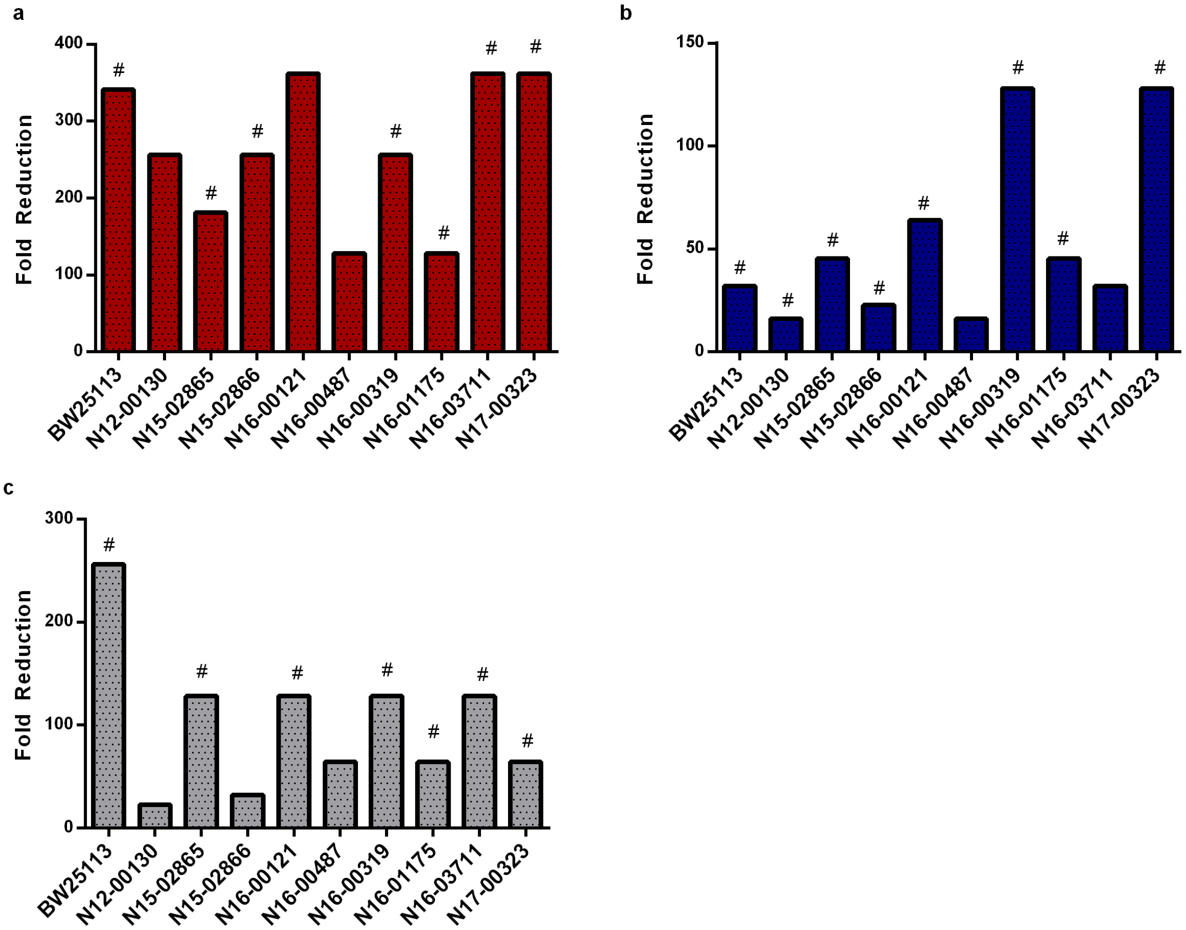

Supplementary Figure 1. Clinical and retail food derived *mcr-1* expressing *E. coli* isolates are susceptible to colistin potentiation. a,b,c, Mean fold reduction of rifampicin (**a**), novobiocin (**b**), and clarithromycin (**c**) in the presence of 2 μg mL^{-1} colistin for nine clinical and retail food derived *mcr-1* positive *E. coli* isolates and *E. coli* BW25113 expressing pGDP2:*mcr-1*. Potentiation below clinical breakpoint is indicated with a #. Data in **a, b,** and **c** are representative of two biological replicates.

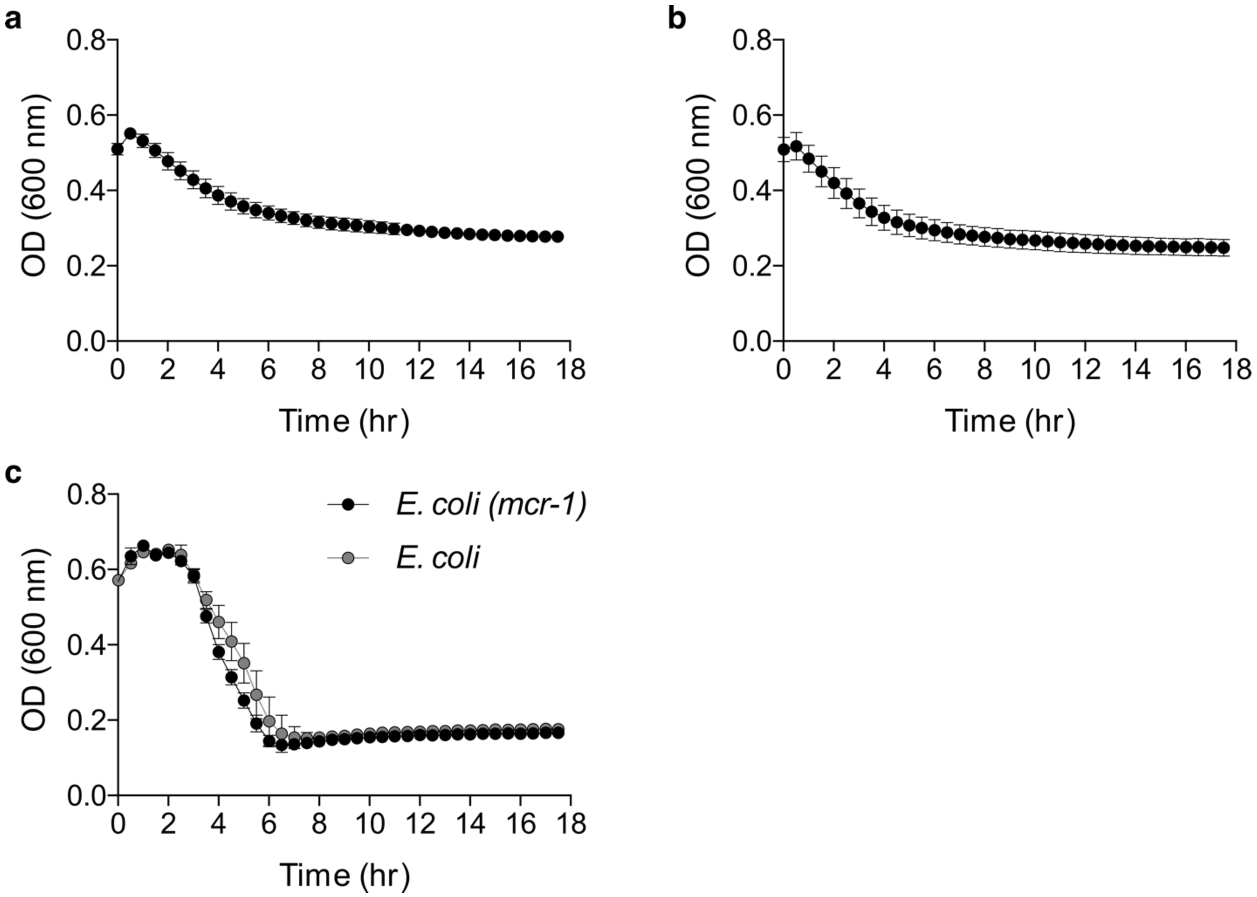

Supplementary Figure 2. Expression of *mcr-1* reduces susceptibility to the lytic activity of colistin. a, b, Kinetic analysis of lysis in *mcr-1* expressing *E. coli* at 100 µg mL^{-1} (**a**) and 200 µg mL^{-1} (**b**) of colistin. **c,** Kinetic analysis of lysis in *mcr-1* expressing (black) and wild type (grey) *E. coli* at 500 µg mL^{-1} ampicillin. **a**, **b**, **c**, OD (600 nm) was monitored every 30 min for 18 hours in the presence of antibiotic and data represents the means with standard deviation from two biological replicates.

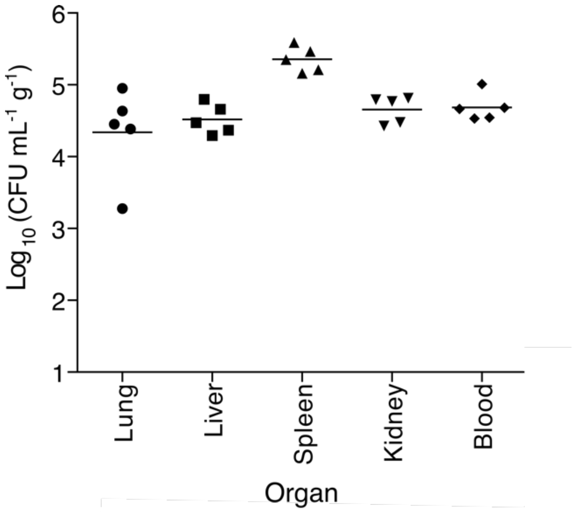

Supplementary Figure 3. ***K. pneumoniae* expressing *mcr-1* demonstrates rapid dissemination in a murine bacteremia model**. Female CD-1 mice (n=5) were infected with *K. pneumoniae* (~1 x 10^6 CFU, i.p.) in PBS and 5% porcine mucin. Mice were euthanized one hour after infection, and a variety of organ tissue aseptically collected weighed, homogenized, serially diluted in PBS and plated onto solid LB supplemented with kanamycin (50 µg mL^{-1}). Plates were incubated overnight at 37 °C and colonies were quantified to determine bacterial load. Horizontal lines represent the geometric mean of the bacterial load for each organ.

Supplementary Table 1. **Minimum inhibitory concentration (MIC) of Enterobacteriaceae strains transformed with pGDP2:*mcr-1* in the presence and absence of 2 μg mL^{-1} colistin.** Columns 1, wild type MIC. Column 2, *mcr-1* expressing MIC. Column 3, MIC of the *mcr-1* expressing strain in the presence of 2 μg mL^{-1} colistin. Column 4, fold reduction determined by dividing column 2 by column 3. Data represent the mean of at least two biological replicates

	E.coli				***E. cloacae***				***E. aerogenes***				***S*. Typhimurium**				***K. pneumoniae***			
**Antibiotic**	**1**	**2**	**3**	**4**	**1**	**2**	**3**	**4**	**1**	**2**	**3**	**4**	**1**	**2**	**3**	**4**	**1**	**2**	**3**	**4**
Azithromycin	12.5	6.25	0.03	181	35.36	25	0.2	128	12.5	6.25	0.2	32	12.5	6.25	0.2	32	6.25	12.5	0.20	64
Clarithromycin	50	50	0.2	256	150	100	0.2	512	150	50	0.1	512	100	50	0.39	128	100	50	0.10	512
Erythromycin	50	50	0.78	64	>200	200	1	200	>200	100	0.28	362	100	100	1.56	64	50	100	0.78	128
Minocycline	2.21	3.13	0.2	16	6.25	3.13	0.2	16	6.25	3.13	0.39	8	4.42	6.25	0.20	32	3.13	3.13	0.39	8
Mupirocin	50	50	0.78	64	100	50	0.39	128	100	50	0.78	64	100	100	0.29	341	50	100	0.39	256
Novobiocin	25	25	0.59	43	>200	>200	1.56	256	25	25	0.59	43	100	>200	1.56	256	50	50	1.56	32
Rifabutin	4.42	4.69	0.01	384	8.84	12.5	0.01	1365	8.84	12.5	0.01	1024	6.25	4.69	0.02	256	6.25	12.5	0.01	1024
Rifampicin	6.25	6.25	0.02	342	12.5	25	0.01	2048	12.5	12.5	0.02	512	12.5	12.5	0.05	256	12.5	12.5	0.02	512
Rifaximin	12.5	12.5	0.05	256	12.5	12.5	0.02	512	12.5	12.5	0.05	256	12.5	6.25	0.10	64	25	12.5	0.05	256
Roxithromycin	>200	200	0.78	256	>200	>200	0.78	512	>200	>200	0.78	512	>200	200	3.13	64	200	>200	0.78	384

Supplementary Table 2. Characterization of spontaneous colistin and rifampicin combination suppressor mutants in *E. coli* BW25113 transformed with pGDP2*:mcr-1*.

	MIC		MIC (Colistin 2 μg mL^{-1})		
**Mutant Strain**	**Colistin (μg mL^{-1})**	**Rifampicin (μg mL^{-1})**	**Rifampicin (μg mL^{-1})**	**Clarithromycin (μg mL^{-1})**	**Novobiocin (μg mL^{-1})**
1	6.25	>200	>200	0.1	3
2	6.25	>200	37.5	0.1	3
3	6.25	>200	>200	0.1	3
4	6.25	>200	>200	0.1	2.25
5	6.25	>200	>200	0.1	1.5
6	6.25	>200	>200	0.1	3
7	6.25	100	50	0.1	1.5
8	6.25	>200	>200	0.4	1.5
9	6.25	>200	>200	0.1	1.5
BW (*mcr-1*)	6.25	6.25	0.02	0.1	1.5
